# Increased Detection of Carbapenemase-Producing Enterobacterales Bacteria in Latin America and the Caribbean during the COVID-19 Pandemic

**DOI:** 10.3201/eid2811.220415

**Published:** 2022-11

**Authors:** Genara Romero Thomas, Alejandra Corso, Fernando Pasterán, Justina Shal, Aldo Sosa, Marcelo Pillonetto, Renata Tigulini de Souza Peral, Juan Carlos Hormazábal, Pamela Araya, Sandra Yamile Saavedra, Mariá Victoria Ovalle, María Antonieta Jiménez Pearson, Grettel Chanto Chacón, Eric Carbon, Carmen Julia Mazariegos Herrera, Selene del Carmen González Velásquez, Carolina Satan-Salazar, Fernando Villavicencio, Nancy Melgarejo Touchet, Sofía Busignani, Maritza Mayta-Barrios, Juan Ramírez-Illescas, Mariana López Vega, Cristina Mogdasy, Verónica Rosas, Nuris Salgado, Rodolfo Quiroz, Nathalie El-Omeiri, Marcelo Fabián Galas, Pilar Ramón-Pardo, Roberto Gustavo Melano

**Affiliations:** Pan American Health Organization, Washington, DC, USA (G. Romero Thomas, R. Quiroz, N. El-Omeiri, M.F. Galas, P. Ramón-Pardo, R.G. Melano);; Instituto Nacional de Enfermedades Infecciosas–ANLIS “Dr. C.G. Malbrán,” Argentina (A. Corso, F. Pasterán);; Central Medical Laboratory, Belize City, Belize (J. Shal, A. Sosa);; Laboratório Centrais de Saúde Pública do Paraná, Curitiba-PR, Brazil (M. Pillonetto);; General Coordination of Public Health Laboratories, Brasilia-DF, Brazil (R. Tigulini de Souza Peral);; Instituto de Salud Pública, Santiago, Chile (J.C. Hormazábal, P. Araya);; Instituto Nacional de Salud, Bogotá, Colombia (S.Y. Saavedra, M.V. Ovalle);; Instituto Costarricense de Investigación y Enseñanza en Nutrición y Salud, Cartago, Costa Rica (M.A. Jiménez Pearson, G. Chanto Chacón);; Princess Margaret Hospital/Dominica-China Friendship Hospital Laboratory, Roseau, Dominica (E. Carbon);; Laboratorio Nacional de Salud, Bárcena, Guatemala (C.J. Mazariegos Herrera);; Unidad Central de Referencia Vigilancia Epidemiológica, Bárcena (S.C. González Velásquez);; Instituto Nacional de Investigación en Salud Pública, Quito, Ecuador (C. Satán-Salazar, F. Villavicencio);; Laboratorio Central de Salud Pública, Asunción, Paraguay (N. Melgarejo Touchet, S. Busignani);; Instituto Nacional de Salud, Lima, Peru (M. Mayta-Barrios, J. Ramírez-Illescas);; Departamento de Laboratorios de Salud Pública, Montevideo, Uruguay (M. López Vega, C. Mogdasy);; Instituto Nacional de Higiene “Rafael Rangel”, Caracas, Venezuela (V. Rosas, N. Salgado);; Public Health Ontario Laboratory, Toronto, Ontario, Canada (R.G. Melano)

**Keywords:** COVID-19, respiratory infections, severe acute respiratory syndrome coronavirus 2, SARS-CoV-2, SARS, coronavirus disease, zoonoses, viruses, coronavirus, antimicrobial resistance, bacteria, carbapenemase-producing Enterobacterales, enteric infections, food safety, Latin America, the Caribbean, *Suggested citation for this article*: Romero Thomas G, Corso A, Pasterán F, Shal J, Sosa A, Pillonetto M, et al. Increased detection of carbapenemase-producing Enterobacterales bacteria in Latin America and the Caribbean during the COVID-19 pandemic. Emerg Infect Dis. 2022 Nov [*date cited*]. https://doi.org/10.3201/eid2811.220415

## Abstract

During 2020–2021, countries in Latin America and the Caribbean reported clinical emergence of carbapenemase-producing Enterobacterales that had not been previously characterized locally, increased prevalence of carbapenemases that had previously been detected, and co-production of multiple carbapenemases in some isolates. These increases were likely fueled by changes related to the COVID-19 pandemic, including empirical antibiotic use for potential COVID-19–related bacterial infections and healthcare limitations resulting from the rapid rise in COVID-19 cases. Strengthening antimicrobial resistance surveillance, epidemiologic research, and infection prevention and control programs and antimicrobial stewardship in clinical settings can help prevent emergence and transmission of carbapenemase-producing Enterobacterales.

Since the beginning of the COVID-19 pandemic, the emergence of resistant microorganisms causing healthcare-associated infections has been documented (*1*). Using antimicrobial drugs in patients with COVID-19 for the treatment of potential, but untested, bacterial pathogens has become a widely implemented empirical practice (*2**–**6*). However, the rapid rise in the number of COVID-19 cases has overwhelmed healthcare systems, producing a multifactorial problem. For example, the shortage of healthcare workers struggling to provide timely care to patients, complications in implementing infection control practices, and the transfer of critically ill patients between hospitals to receive life-saving care have increased the potential emergence and dissemination of resistant bacteria in places where they were not previously identified. Hospitalization predisposes patients, including those with COVID-19, to healthcare-associated or secondary infections, especially in intensive care units (ICUs). This situation was observed in US hospitals, where increased prevalence of carbapenemase-producing microorganisms was aggravated by a higher rate of device-associated infections in ICUs, mainly because of central vascular catheters and mechanical ventilation (*7*). This complex situation could be associated with an increase in deaths attributable to infections by multidrug-resistant (MDR) microorganisms, as was demonstrated before the COVID-19 pandemic (*8*,*9*). Antimicrobial resistance (AMR) will continue to be a major public health problem that will most likely intensify in the near future.

The emergence of gram-negative pathogens resistant to carbapenem antibiotics is one of the most pressing clinical problems worldwide, particularly when the pathogens produce β-lactamases that have full hydrolyzing activity against carbapenems (*10*–*12*). Since the description of the first carbapenemase, several of these enzymes, including *Klebsiella pneumoniae* carbapenemase (KPC), oxacilinase (OXA)-48–like carbapenemase, New Delhi metallo-β-lactamase (NDM), Verona integron–encoded metallo-β-lactamase (VIM), and imipenemase (IMP), have been disseminated globally and are commonly detected in Enterobacterales, *Pseudomonas* spp., and *Acinetobacter* spp.

Some of these enzymes emerged in Enterobacterales bacteria and produced rapid endemicity (*13*) or caused large hospital outbreaks (*14*). Of note, certain countries and regions had a higher frequency of detection than others (*15*), including sporadic descriptions of isolates of Enterobacterales co-producing >2 types of the most common carbapenemases (*16*,*17*). Here, we summarize the increased detection of carbapenemase-producing Enterobacterales (CPE) detected and reported by the national AMR surveillance networks in Latin America and the Caribbean coincident with the COVID-19 pandemic.

## Situation in Latin America and the Caribbean before the COVID-19 Pandemic

The Latin American Network for Antimicrobial Resistance Surveillance (ReLAVRA [Spanish acronym], https://www.paho.org/en/relavra) was established by the Pan American Health Organization/World Health Organization (PAHO/WHO) in 1996. Its goal is to inform AMR prevention and control policies and interventions in the region through ongoing collection of reliable, comparable, and reproducible AMR data focused on resistance surveillance of community-acquired and nosocomial pathogens. The network currently consists of 33 countries, 19 from Latin America and, since 2018, another 14 from the Caribbean; the organization was renamed ReLAVRA+ to note the additional countries. These countries are represented by their National Reference Laboratories (NRL), officially designated by the national authority in each country. Each NRL, head of the national network, is responsible for external quality control for all participating laboratories, routinely ensuring the reliability of the tests performed, including the validity of species identification and characterization, antibiotic susceptibility tests, and data quality. Methods used are determined according to the complexity of each NRL but are common for carbapenem-resistant Enterobacterales (CRE) detection in general. Antibiotic susceptibility testing is the first step, followed by phenotypic confirmation of carbapenemase activity in CRE. Testing includes disc-inhibitor synergy tests, blue-carba test, carba-NP test, or modified carbapenem inactivation method, followed by molecular methods (mainly PCR) for carbapenemase gene confirmation. Depending on the country’s epidemiology, all CRE samples are submitted to the NRLs for carbapenemase confirmation when these microorganisms are emerging in the country or their detection is relatively low; in countries where CPEs are endemic, only a proportion of these pathogens (e.g., first isolates of each sentinel hospital) are submitted according to changes in their epidemiology, emergence of new carbapenemase combinations, or new AMR such as ceftazidime/avibactam resistance. These surveillance data are reported to and aggregated by ReLAVRA+, which has been monitoring carbapenem resistance in gram-negative bacilli for >15 years (*18*). For example, resistance to carbapenems in *K. pneumoniae* was a sporadic finding among some reporting countries in the region during 2006–2010. During 2010–2019, countries reported a slow but sustained increase in meropenem nonsusceptibility (intermediate plus resistance categories) and a wide heterogeneity in its magnitude, which reached high percentages of >60% in some countries (Figure). This situation was also observed in comprehensive literature reviews of the epidemiology of these enzymes in Latin America and the Caribbean during 2013–2019 (*19*,*20*). These publications described the rapid and successful dissemination of the *bla*_KPC_ carbapenemase gene in Enterobacterales throughout the region and overt endemicity in countries such as Brazil, Colombia, and Argentina (*21*). The presence of other carbapenemase genes, such as *bla*_NDM_ and, to a lesser extent, *bla*_IMP_ and *bla*_VIM_, were also described during 2013–2016. Enterobacterales bacteria carrying a combination of these genes occurred in isolated cases, including *bla*_NDM_ + *bla*_KPC_ (n = 3) and *bla*_VIM_ + *bla*_KPC_ (n = 1) in *K. pneumoniae* from Colombia, *bla*_VIM_ + *bla*_KPC_ (n = 1) in *Enterobacter cloacae* from Venezuela, and *bla*_NDM_ + *bla*_KPC_ (n = 2) in *Enterobacter* spp. from Brazil (*19*). During 2016–2019, just before the pandemic, an increase in *bla*_NDM_ gene detection was observed in Enterobacterales from different countries in Latin America (*20*). However, description of carbapenemase gene combinations before the onset of the COVID-19 pandemic were limited in Enterobacterales: *bla*_VIM_ + *bla*_KPC_ (n = 6) and *bla*_KPC_ + *bla*_NDM_ (n = 3) from Colombia, *bla*_NDM_ + *bla*_KPC_ (n = 4) in *K. pneumoniae* from Brazil, an outbreak of *K. pneumoniae* ST833 carrying *bla*_VIM_ + *bla*_KPC_ from Venezuela, and *bla*_NDM_ and an unknown *bla*_IMP_ variant (n = 1) in a *Providencia rettgeri* isolate from Mexico (*20*).

**Figure:**
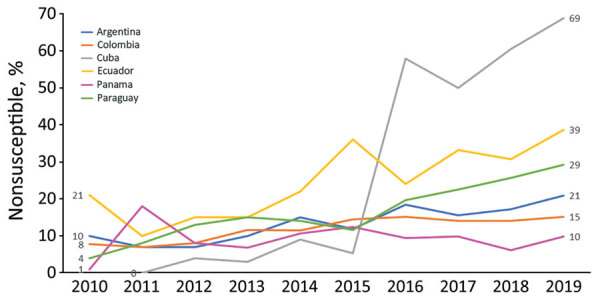
Increased rates of meropenem nonsusceptibility (intermediate plus resistance categories) in *Klebsiella pneumoniae* from countries reporting changes in the prevalence of some carbapenemase genes in study of increased detection of carbapenemase-producing Enterobacterales bacteria in Latin America and the Caribbean during the COVID-19 pandemic. Countries listed are those that had continuous susceptibility data in the Latin American Network for Antimicrobial Resistance Surveillance database.

## Situation during the COVID-19 Pandemic

Several NRLs in Latin America (ReLAVRA+ member countries) have issued national alerts highlighting the emergence of carbapenemase genes not described before in Enterobacterales, increases in isolates positive for previously reported carbapenemase genes, or isolates co-harboring >2 of these genetic determinants. This regional situation triggered the publication of an epidemiologic alert by PAHO/WHO (*22*).

Argentina reported this increase in an epidemiologic alert in April 2021 (*23*). A gene combination not previously documented in the prepandemic years, *bla*_KPC_ + *bla*_NDM_, was found mainly in *K. pneumoniae* isolated from 8 hospitals (Table). Further studies with a larger collection of *K. pneumoniae* positive for *bla*_KPC_ + metallo-β-lactamase activity (May 2020–June 2021; 77 isolates from 27 hospitals) revealed that most bacteria carried *bla*_KPC_ + *bla*_NDM_ and only a few carried *bla*_KPC_ + *bla*_IMP_. Pulse-field gel electrophoresis showed a high diversity of 36 XbaI types among the 77 isolates, suggesting independent acquisition of those *K. pneumoniae* strains in Argentina. Since then, other countries in the region have also published epidemiologic alerts, such as Guatemala (*24*,*25*), or reported the detection of isolates positive for double carbapenemase genes, including Paraguay (*26*) and Brazil (*27*) (Table).

**Table:** Summary of findings in countries reporting changes in the prevalence of some carbapenemase genes in study of increased detection of carbapenemase-producing Enterobacterales bacteria in Latin America and the Caribbean during the COVID-19 pandemic*

Country	Period	Findings
Argentina, Instituto Nacional de Enfermedades Infecciosas–ANLIS “Dr. C.G. Malbrán”	Pandemic, May 2020–Jun 2021	First detection of double carbapenemase genes (52/196), 60% of which harbored *bla*_KPC_ + *bla*_NDM_ (mainly in *Klebsiella pneumoniae* 31/32). Further studies of *K. pneumoniae* (n = 77) revealed 74/77 isolates with *bla*_KPC_ + *bla*_NDM_ and 3/77 isolates with *bla*_KPC_ + *bla*_IMP_.
Brazil, Laboratório Centrais de Saúde Pública do Paraná, LACEN-PR	Prepandemic, Jul 2017–Feb 2019	CRE (n = 3,712). Carbapenemase genes detected: 73.6% (2,731/3,712); isolates carrying *bla*_KPC_ + *bla*_NDM_: 2% (55/2,731).
	Pandemic, Mar 2020–Oct 2021	CRE (n = 5,300), increase of 42.8%. Carbapenemase genes detected: 88.4% (4,683/5,300); isolates carrying *bla*_KPC_ + *bla*_NDM_: 4.7% (219/4,683). *K. pneumoniae* represented 49% (107/219) of bacteria.
Colombia, Instituto Nacional de Salud	Prepandemic, Jan 2018–Dec 2019	CPE (n = 262); isolates carrying *bla*_KPC_ + *bla*_NDM_ + *bla*_VIM_: 1.1% (3/262); first detection in 2018.
	Pandemic, Jan 2020–Dec 2021	CPE (n = 557), increase of 112.6%. Isolates carrying *bla*_KPC_ + *bla*_NDM_ + *bla*_VIM_: 3.6% (20/557).
Uruguay, Departamento de Laboratorios de Salud Pública	Prepandemic, Jan 2017–Dec 2019	<1% (5/530) of *Enterobacterales* carrying *bla*_KPC_ + *bla*_NDM_.
	Pandemic, Jan 2020–Jun 2021	5.6% (18/321) of *Enterobacterales* carrying *bla*_KPC_ + *bla*_NDM_.
Guatemala, Laboratorio Nacional de Salud	Pandemic, Nov 2020	First detection of *bla*_OXA-48_–like (*bla*_OXA-232_ and *bla*_OXA-181_) in *K. pneumoniae* (n = 4) and *Escherichia coli* (n = 1) confirmed by whole-genome sequencing.
	Pandemic, Apr 2021	First detection of *bla*_KPC_ + *bla*_NDM_ in *Enterobacter cloacae* complex (n = 2).
Paraguay, Laboratorio Centra de Salud Pública	Pandemic, Feb–Sep 2021	First detection of *bla*_KPC_ + *bla*_NDM_ in *K. pneumoniae* (n = 6) and *bla*_OXA-48_-like + *bla*_NDM_ in *E. cloacae* (n = 1)).
Peru, Instituto Nacional de Salud	Pandemic, Jul–Oct 2021	First detection of *bla*_KPC_ + *bla*_NDM_ in *K. pneumoniae* (n = 2) and *bla*_OXA-48_–like + *bla*_NDM_ in *E. coli* (n = 1).
Ecuador, Instituto Nacional de Investigación en Salud Pública-“Dr. Leopoldo Izquieta Pérez”	Pandemic, Jan–Feb 2021	First detection of *bla*_KPC_ + *bla*_NDM_ in *K. pneumoniae* (n = 1) and *bla*_OXA-48_–like + *bla*_NDM_ in *E. coli* (n =1).
Venezuela, Instituto Nacional de Higiene “Rafael Rangel”	Pandemic, Oct 2021	First detection of *bla*_KPC_ + *bla*_NDM_ in *K. pneumoniae* (n = 1).
Costa Rica, Instituto Costarricense de Investigación y Enseñanza en Nutrición y Salud	Pandemic, Dec 2021	First detection of *bla*_IMP_ + *bla*_NDM_ in *E. cloacae* complex (n = 1).
Belize, Central Medical Laboratory	Pandemic, Jan–May 2021	First detection of *bla*_NDM_ in *K. pneumoniae* (n = 4) and *E. coli* (n = 2).
Dominica, Princess Margaret Hospital Medical Laboratory	Pandemic, Dec 2020–Mar 2021	First detection of *bla*_NDM_ in* K. pneumoniae* (n = 2) and* E. coli* (n = 1).
Chile, Instituto de Salud Pública	Pandemic, Apr–Jul 2021	First detection of *bla*_OXA-48_–like in *K. pneumoniae* (n = 22) and *E. coli* (n = 1).

*CPE, carbapenemase-producing Enterobacterales; CRE, carbapenem-resistant Enterobacterales; *bla*, β-lactamase gene; KPC, *Klebsiella pneumoniae* carbapenemase; OXA, oxacillinase; NDM, New Delhi metallo-β-lactamase; VIM, Verona integron–encoded metallo-β-lactamase; IMP, imipenemase.

During the pandemic, the NRLs of Peru, Ecuador, Venezuela, and Costa Rica directly communicated the emergence of carbapenemase combinations to PAHO/WHO, and the emergence of carbapenemase genes not previously detected at the national level was reported in Belize, Dominica, and Chile (Table). Others have reported an increase in CPE prevalence. During January 2020–June 2021, Uruguay reported an increase in Enterobacterales carrying *bla*_KPC_ + *bla*_NDM_ genes. The NRL of Colombia reported an increase in the number of CPE strains in 2021, which coincided with the time of highest hospital occupancy because of COVID-19. Colombia had >2 times the number of CPEs during January 2020–December 2021 than during 2018–2019; most had *bla*_KPC_ (endemic in the country) and *bla*_NDM_ genes. The frequence of the triple combination *bla*_KPC_ + *bla*_NDM_ + *bla*_VIM_, identified in 2018, increased to 3.6% during 2020–2021 (Table). The Regional Public Health Laboratory (LACEN-PR) of Brazil, which receives data from hospitals in 5 states (Rio Grande do Sul, Santa Catarina, Paraná, Mato Grosso do Sul, and Mato Grosso), showed a 42.8% increase in the submission of CRE isolates (confirmed by LACEN-PR) during the pandemic period (March 2020–October 2021; n = 5,300) compared with the 20 months before the pandemic (July 2017–February 2019; n = 3,712). An increase in the total number of detected carbapenemase genes was also observed: 73.6% before the pandemic, compared with 88.4% during the pandemic (Table). Paraná state in Brazil showed the largest increase, although Santa Catarina (56.8% increase) and Rio Grande do Sul (43.5% increase) also contributed to the overall increase in the total number of detected carbapenemase genes. The carbapenemase genes identified most often were *bla*_KPC_ (55.6% increase, mainly in *K. pneumoniae*) and *bla*_NDM_ (116.7% increase). Double carbapenemase gene detection (*bla*_KPC_ + *bla*_NDM_) also increased from 2% in the prepandemic period to 4.7% during the pandemic (Table).

Because of the plasmidic nature of the coding genes of these enzymes and MDR phenotype of these bacteria, the risk of dissemination is increased. The presence of these emerging resistances is aggravated by the co-expression of resistance mechanisms to other therapeutically critical antibiotics in the region, such as polymyxins, limiting the antimicrobial treatment of these pathogens. The dissemination of double carbapenemases is also being observed at the regional level in nonfermenting bacteria, such as *Pseudomonas* spp. and *Acinetobacter* spp., a situation not addressed in this report.

The emergence of carbapenem resistance in Enterobacterales during the pandemic led PAHO/WHO to recommend the implementation and strengthening of surveillance and epidemiologic research to member states. Recommendations were provided to detect the presence of microorganisms carrying carbapenem resistance genes and start timely prevention and control measures, together with adopting effective antimicrobial drug stewardship programs at both the hospital and outpatient levels (*21*).

## Discussion

The overuse of antibiotics, their inappropriate prescribing, and their extensive animal and agricultural use play a role in the evolution of AMR, one of the most troublesome current global health crises. Before the pandemic, alarming numbers related to antibiotic-resistant pathogens were released by the European Centre for Disease Prevention and Control and the US Centers for Disease Control and Prevention: >670,000 infections and 33,000 deaths caused by resistant bacteria were reported in Europe in 2019, whereas, in the United States, >3 million new infections and 35,900 deaths were caused by resistant bacteria and fungi each year (*28*,*29*). Moreover, the projections of AMR burden for the future are worrisome. In a review commissioned by the UK government in 2014, it was estimated that AMR could cause 10 million deaths a year by 2050 (*30*). A recent publication addressing the global burden of AMR estimated a median of 1.27 million deaths directly attributable to resistance and 4.95 million deaths associated with bacterial AMR worldwide in 2019 (*31*).

Those data had not yet considered the effects of the COVID-19 pandemic on antibiotic use, and, perhaps most important, in the management of patients in a pandemic context. Healthcare facilities were faced with limited resources, lack of hospital beds and isolation rooms for COVID-19 cases, increased critical care needs, and shortages of personal protective equipment, and the consequences included incorrect or suboptimal implementation of prevention and infection control measures. Any of those shortages could have contributed to emergence and dissemination of AMR. Those changes, particularly during the first stages of the pandemic, could also affect AMR surveillance. In an international survey conducted by WHO to assess the effects of COVID-19 on AMR surveillance, prevention, and control, a reduction in funding for AMR activities was reported more frequently by low- and middle-income countries (LMICs), affecting the possibilities of acquiring quality laboratory reagents and consumables for bacteriology and antimicrobial susceptibility testing and the ability to service machines and equipment (*32*). These conditions were also reported to ReLAVRA+ by some of the NRLs in Latin America.

As with other coronavirus epidemics, such as those caused by SARS-CoV-1 and Middle Eastern respiratory syndrome viruses, studies suggest that there is no increased rate of bacterial and fungal co-infection with SARS-CoV-2, which has been observed with influenza and other viral diseases (*1*,*33*–*37*). Nevertheless, a large proportion of patients hospitalized with COVID-19 received antibiotics even if the bacterial co-infection rate was low (*33*,*38*–*40*). In Latin America, the empirical use of antibiotics in the context of the pandemic has been documented (*41*,*42*) and worsens the potential for antimicrobial resistance to available drugs. This last point raises another complication. Before the pandemic, most LMICs in Latin America and the Caribbean did not have access to newly developed antibiotics because of their cost or lack of availability, which resulted in increased use of last-resort antibiotics, including old and less safe drugs such as polymyxins, to treat life-threatening bacterial diseases. Point prevalence surveys of antibiotic use conducted in hospitals in different countries in Latin America before the pandemic showed a very high prevalence of antibiotic use, ranging from 33% to 54% (*43*,*44*). In the most recent survey performed during December 2018–August 2019 in 33 hospitals from 5 countries (Cuba, El Salvador, Mexico, Paraguay, and Peru), extended-spectrum cephalosporins were the class of antimicrobial drugs most frequently used (26.8%), followed by carbapenems (10.3%) and fluoroquinolones (8%) (*45*). Antimicrobial drugs were used more frequently in ICUs (67.2%), surgical wards (64.5%), and medical wards (54.2%). Emergence of clinical CPEs during the COVID-19 pandemic that had not previously been characterized locally, the increased prevalence of CPEs that had previously been detected, and detection of Enterobacterales harboring multiple carbapenemase genes was likely the result of following the same or an increased trend of high antibiotic use, particularly the most potent β-lactams, reported in prepandemic years. The pandemic might have accelerated the problem of resistance to the limited antimicrobial arsenal in the region because of antimicrobial drug misuse, prolonged hospital stays, and a high rate of antimicrobial prescriptions in hospital settings, such as medical wards and ICUs, where a high number of patients with COVID-19 were hospitalized during the first pandemic wave. Updated regional data about the use of antimicrobial drugs during the pandemic are required to confirm these assumptions. For instance, more than half of the responding countries in a WHO survey reported increases in total prescriptions of antibiotics during the COVID-19 pandemic (*32*).

Emergence of antimicrobial-resistant pathogens in a region without access to new antibiotics is of great concern. A recent publication indicated that high-income countries, such as Canada, Japan, France, Germany, Italy, and Spain, have limited access to newly approved antibiotics, including ceftazidime/avibactam, cefiderocol, imipenem-cilastatin/relebactam, meropenem/vaborbactam, and ceftolozane/tazobactam, which suggests that pharmaceutical companies might have decided to delay commercialization because insufficient profits were expected (*46*). That publication highlights the complications of providing patient access to those drugs only in countries with broad economic resources and suggests that making these drugs available in LMICs is currently difficult.

Detection and identification of carbapenem-resistance mechanisms reflect the improvements made in laboratory capacity in the region during the prepandemic years that ensured their timely detection and characterization. PAHO/WHO’s Latin American Program for Quality Control in Bacteriology and Antimicrobial Resistance, coordinated by the Regional Reference Laboratory (Servicio Antimicrobianos, INEI-ANLIS “Dr. C. G. Malbrán”), was built by more than 2 decades of intensive work in the region (including NRLs of 17 countries: Bolivia, Chile, Colombia, Costa Rica, Ecuador, El Salvador, Guatemala, Honduras, Cuba, Mexico, Nicaragua, Panama, Paraguay, Peru, Dominican Republic, Uruguay, and Venezuela). This network has a strong supportive interaction between NRLs, a critical feature for a region composed of LMICs. The performance of this regional network was documented, showing continuous quality improvement in the diagnosis of infections caused by MDR microorganisms (*47*). With the same objectives in mind, the Caribbean External Quality Assurance Program in Bacteriology and Antimicrobial Resistance was implemented in 2018; the current participating NRLs are from 12 countries: Belize, Barbados, Suriname, Antigua and Barbuda, Dominica, Grenada, Saint Lucia, Saint Kitts and Nevis, Saint Vincent and the Grenadines, Haiti, Guyana, and Trinidad and Tobago. Those regional networks are fundamental to continue monitoring antimicrobial resistance emergence, vital not only for preventing their dissemination but for ensuring continuous effectiveness of available antimicrobial drugs. In addition, the NRLs in Latin America, coordinated by PAHO/WHO, have instituted a regional consensus to standardize definitions for different levels of antimicrobial resistance in 3 of the main gram-negative bacteria of public health importance (*K. pneumoniae*, *Pseudomonas aeruginosa*, and *Acinetobacter* spp.) and for identification and constant, unified surveillance of these resistant microorganisms according to regional definitions (e.g., type of antibiotics accessible in the countries, different capacities of the countries involved, and local resistance epidemiology) (*48*).

Even with the support of the Regional Reference Laboratory and the horizontal collaborations between NRLs, one of the main limitations of this study is that we do not have updated reports from all countries in the region. Some countries had to stop AMR surveillance because of lack of reagents and personnel during the COVID-19 pandemic. Another limitation is the lack of implementation of molecular techniques for identifying carbapenem-resistance mechanisms in some LMICs because they do not have the equipment or are unable to acquire reagents that are often too expensive. Nevertheless, phenotypic identification has been implemented, but the epidemiology of circulating CPEs remains unknown.

In conclusion, AMR has become a catastrophic public health and economic problem because of the emergence of new resistance mechanisms and lack of new antimicrobial drugs. The onset of the COVID-19 pandemic has likely accelerated AMR because of inappropriate use of antibiotics and inadequate infection prevention and control practices. AMR mechanisms that have emerged during the pandemic, such as carbapenem resistance, might be the result of these unfavorable changes, further reinforcing the need for new antimicrobial drug access in LMICs. The COVID-19 pandemic has also shown the inequities between countries for access to vital public health tools, as mentioned in a recent publication: “Just as COVID-19 has stimulated a movement to re-balance inequitable access between high- and LMICs, it’s time that mitigating antibiotic resistance got a healthy dose of the same” (*49*). In an ideal scenario, access to newly developed and approved antimicrobials and tools to guide their prudent use should be guaranteed for the entire world in parallel with current local AMR prevention and control strategies, including the prioritization of antimicrobial stewardship and rapid AMR detection. These measures are particularly critical in a region where MDR Enterobacterales bacteria that coproduce the most potent carbapenemases (NDM and KPC) are broadly emerging.
